# High-Risk Cardiovascular Patients: Clinical Features, Comorbidities, and Interconnecting Mechanisms

**DOI:** 10.3389/fimmu.2015.00591

**Published:** 2015-11-23

**Authors:** Katharina Andrea Schuett, Michael Lehrke, Nikolaus Marx, Mathias Burgmaier

**Affiliations:** ^1^Department of Internal Medicine I, University Hospital RWTH Aachen, Aachen, Germany

**Keywords:** atherosclerosis, type 2 diabetes mellitus, systemic lupus erythematosus, rheumatoid arthritis, coagulation, inflammation, cardiovascular disease

## Abstract

Cardiovascular disease is the leading cause of death in the Western world with an increase over the last few decades. Atherosclerosis with its different manifestations in the coronary artery tree, the cerebral, as well as peripheral arteries is the basis for cardiovascular events, such as myocardial infarction, stroke, and cardiovascular death. The pathophysiological understanding of the mechanisms that promote the development of vascular disease has changed over the last few decades, leading to the recognition that inflammation and inflammatory processes in the vessel wall are major contributors in atherogenesis. In addition, a subclinical inflammatory status, e.g., in patients with diabetes or the presence of a chronic inflammatory disease, such as rheumatoid arthritis, have been recognized as strong risk factors for cardiovascular disease. The present review will summarize the different inflammatory processes in the vessel wall leading to atherosclerosis and highlight the role of inflammation in diabetes and chronic inflammatory diseases for cardiovascular morbidity and mortality.

## Introduction

Cardiovascular disease is the leading cause of death in the western world with an increase over the last few decades (1–4).

Atherosclerosis may result in myocardial infarction (MI), stroke, or peripheral artery disease according to its manifestations in the coronary artery tree, in cerebral arteries as well as peripheral arteries. The pathophysiological understanding of the mechanisms that promote the development of vascular disease has changed over the last few decades, leading to the recognition that inflammation and inflammatory processes in the vessel wall are major contributors in atherogenesis (5).

## Atherogenesis

For decades, atherogenesis has been seen as a degenerative process in the vessel wall leading to the progressive occlusion of the artery up to a certain point where a few activated platelets are sufficient to occlude the vessel and cause cardiac events, such as acute MI. In the 1990s, we learned from our pathology colleagues that a majority of lesions causing an acute coronary syndrome show a stenosis <50%, and further pathological analysis revealed that most of these lesions are the so-called unstable lesions (6). These lesions are characterized by a high inflammatory burden of cells, such as monocytes and T cells, and a large necrotic lipid core as well as a very thin fibrous cap making the atherosclerotic lesion prone to rupture. At the same time, experimental data have shown that atherogenesis is an inflammatory process in the vessel wall in different phases and stages (7). Also not strictly defined endothelial dysfunction, fatty streak formation and the formation of advanced and potentially complicated lesions have been described as the main phases in lesion development (8). Over the last decade, our understanding of the inflammatory cells involved in these processes has grown. The characteristic cell type of an atherosclerotic lesion is the lipid-loaden macrophage which is attracted by certain chemokines to the vessel wall and then can contribute to the local inflammatory response by the expression and release of inflammatory mediators. The uptake of LDL in these mononuclear cells by scavenger receptors results in the characteristic foam cell. In addition, the accumulation of cholesterol within macrophages leads to crystal formation, and these intracellular microcrystals can activate the inflammasome of the cells subsequently resulting in the release of cytokines (9). Interestingly, experimental work from various groups around the world has shown that different subtypes of macrophages can be found in atherosclerotic lesions with the predominance of the M1-subpopulation which release proinflammatory cytokines (10). Additional cellular components of the innate immune response in the vessel wall are mast cells and to a certain extent polymorphonuclear leukocytes, but the latter have mainly been found in murine models, and the relevance for human atherosclerosis remains unclear (11). In addition to the innate immune response, clinical and experimental data support the recognition that the adaptive immune system is of critical importance in atherogenesis. As such, dendritic cells have been identified as a critical “link” between the innate and adaptive immune responses by initiating T-cell response. T cells in the atherosclerotic lesion are mainly CD4 cells which to a large extent release proinflammatory Th1 cytokines. These cytokines, such as interferon (IFN)-γ and tumor necrosis factor (TNF)-α, do not only induce the expression of certain chemokines thus favoring the recruitment of additional leukocytes but also lead to the activation of other vascular cells [reviewed in Ref. (12)].

In addition, recent work has shown that so-called regulatory T cells are an important atheroprotective cell population in the vascular wall through their capacity to control effector T cells (13).

The recognition that inflammation plays an important role in atherogenesis has been paralleled by the clinical observation that elevated levels of high-sensitive C-reactive protein (hsCRP) is a useful biomarker to predict cardiovascular events in patients (14). Elevated levels of hsCRP are associated with an increased risk for MI and major cardiovascular events in a primary prevention population. In addition, a clinical trial with stratification of patients according to their CRP levels has shown that the implementation of a lipid lowering therapy by statins is of greatest benefit in those subjects that exhibited elevated levels of the inflammatory marker hsCRP (15).

## Coagulation and Inflammation

**Figure 1:**
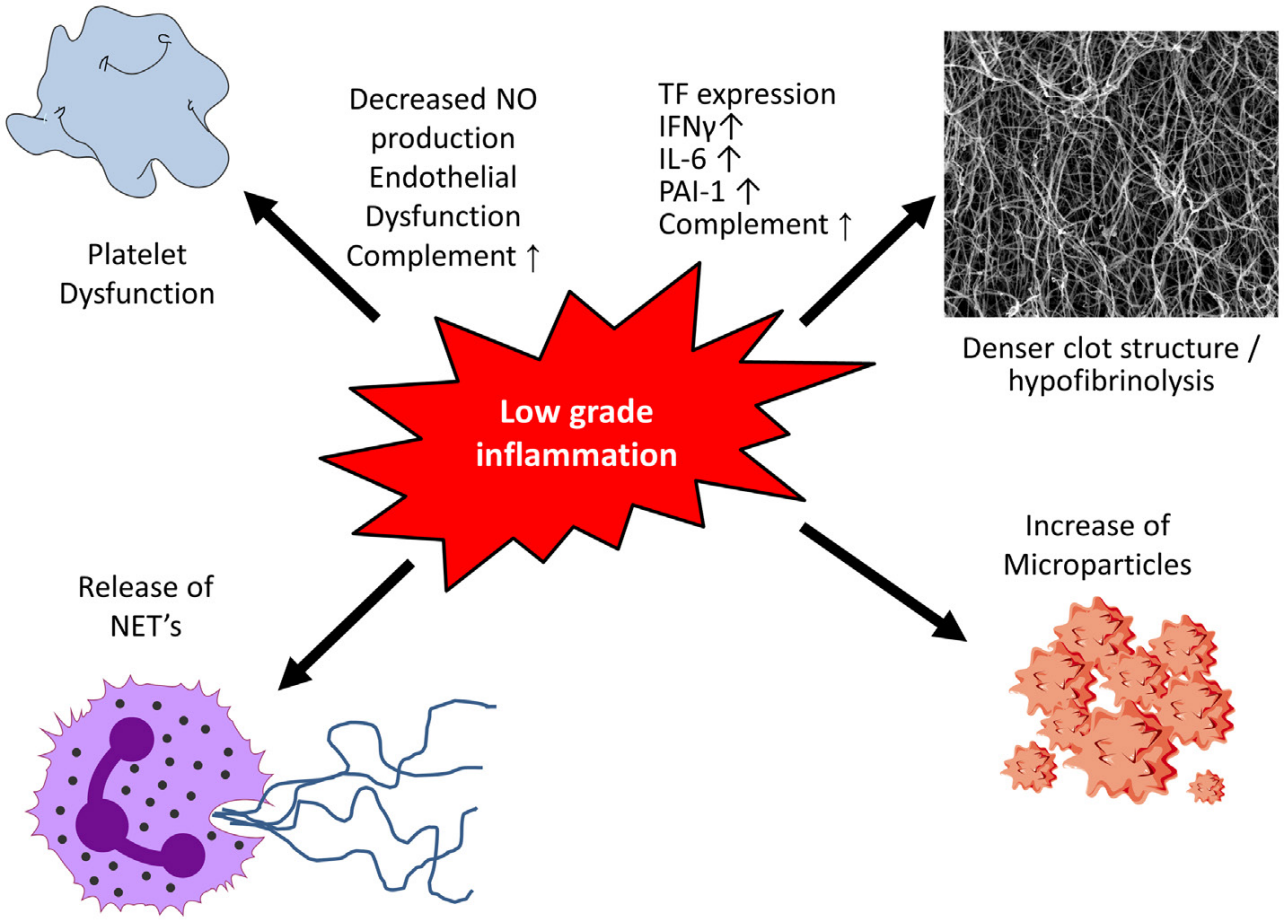
**Low-grade inflammation and coagulation**. Low-grade inflammation results in platelet dysfunction and the formation of a denser clot structure and hypofibrinolysis. Following activation or apoptosis various cell types release microparticles which contain procoagulants. Activation of neutrophils causes the release of neutrophil extracellular traps (NETs), which display procoagulant properties including platelet activation.

Over the last two decades, experimental data have shown an interaction of inflammation with procoagulatory mechanisms in the vessel wall: inflammatory cells, such as macrophages and T lymphocytes, produce cytokines leading to the expression of tissue factor (TF) by endothelial cells, macrophages, and smooth muscle cells (SMCs), thus enhancing prothrombotic properties of the plaque (16). However, the interaction of inflammation and coagulation is not limited to the vessel wall. In obesity, which is frequently associated with cardiovascular disease and diabetes, adipose tissue is a major source for elevated inflammatory cytokines (17, 18). Macrophages and T lymphocytes produce IFN-γ, interleukin (IL)-6, monocyte chemotactic protein (MCP)-1, TNF-α, and PAI-1 (19), which are all able to impact on the coagulation system (Figure 1). Not only inflammatory cytokines but also the complement system interacts with coagulation. The complement system is an important immune surveillance system with over 30 components involved. It can be activated by three different pathways, the classical, the lectin, and the alternative pathway (20). Activation of any of these consequently results in activation of complement C3, the central component of the complement system. Subsequently, complement C5 is activated leading to the formation of the terminal complement complex (C5b, C6, C7, C8, and C9). The biological functions of the complement system are broad including, among others, innate immune responses and coagulation (21). In coagulation, complement affects both platelet function and fibrin clot structure and lysis.

### Primary Hemostasis

Following plaque rupture, thrombosis is initiated when thrombotic components of the plaque are exposed to circulating blood. Platelets are the first to respond and following their adhesion, platelets are activated leading to additional recruitment and aggregation with stabilization of the clot (22). In addition, inflammation can alter platelet function (Figure 1). Early changes include endothelial dysfunction with decreased nitric oxide (NO) production. NO is a key player in negative regulation of platelet activity, and decreased levels result in hyper-reactivity of platelets (23).

In addition to their primary role in hemostasis, platelets actively participate in inflammatory and immune processes. They carry a multiplicity of chemokines and cytokines, which are mostly found within the various platelet granules. Platelet activation and consequent degranulation result in the release of chemokines, including CXC-chemokine ligand 1 (CXCL1), platelet factor 4 (also known as CXCL4), CXCL5 and CXCL7, CC-chemokine ligand 3 [CCL3; also known as macrophage inflammatory protein 1 alpha (MIP-1α)], CCL5 [also known as regulated on activation, normal T Cell expressed and secreted (RANTES)], and CCL7, ILs (IL-1β, IL-7, and IL-8), prostaglandins, and the transmembrane protein CD154 (24, 25). CD154 interacts with CD40 on endothelial cells leading to an upregulation of cell adhesion molecules on endothelial cells, including intercellular adhesion molecule 1 (ICAM1) and vascular cell adhesion molecule 1 (VCAM1), and the release of CC-chemokine ligand-2 (CCL2), thereby facilitating leukocyte recruitment to the inflammatory site (26). In addition, activated platelets also release soluble CD154 likewise leading to an upregulation of vascular cell adhesion molecules and the release of IL-6. Through the expression of these chemokines, cytokines, CD154, and cell adhesion molecules, activated platelets promote neutrophil tethering and activation. Furthermore, they also activate monocytes and dendritic cells, particularly through the CD154–CD40 interaction (24). For an extended overview about this topic, see the review article of Semple et al. (24).

Complement and platelets interact on different levels. Platelets contain several complement receptors as well as complement regulatory molecules, and several components of the complement cascade have been recognized on the platelet surface. Complement activation can be initiated by several platelet agonists, such as adenosindiphosphate (ADP), epinephrine, arachidonic acid, thrombin, and exposure to shear stress (27). However, a study using complement C3 knock-out mice revealed C5 convertase activity of thrombin suggesting a platelet-independent mechanism of complement activation (28). The other way round, several complement components are known to activate platelets including the mannose-binding lectin (MBL)/MBL-associated serine protease (MASP) complex, binding of C1q to its receptor as well as the complement C3 cleavage products (iC3b, C3d, and C3dg) to their receptors CR2 and CR3 (only for iC3b) (27, 29–31). *In vitro* studies further suggest that activated platelets bind the anaphylatoxins C3a and C5a resulting in platelet aggregation (32, 33). In addition to classical complement receptors, P-selectin binds C3b resulting in the generation of C3a and the terminal complement complex C5b–9, thereby enhancing platelet response (34, 35). Furthermore, the anaphylatoxins C3a and C5a are generated which are known to have cytokine-like properties with enhanced leukocyte recruitment and support of the general inflammatory response (27). Under normal circumstances, complement activation is tightly regulated due to expression of complement regulatory molecules. On the platelet surface, factor H inactivates C3b and destabilizes the C3 convertase (36). Similarly, membrane cofactor (MCP or CD46) inactivates C3b, and decay-accelerating factor (DAF or CD55) interferes with C3 convertase. Furthermore, CD59 is known to destabilize the terminal complement complex (35).

### Secondary Hemostasis

Fibrin clot structure plays a crucial role in determining predisposition to atherothrombotic events. Clots with compact structure and impaired fibrinolysis are associated with premature and more severe cardiovascular disease.

Inflammatory cytokines produced by macrophages and T lymphocytes, including IFN-γ, IL-6, MCP-1, and TNF-α, are able to influence both clot structure and lysis (Figure 1). By leading to increased production of acute phase products, such as fibrinogen and PAI-1, they alter clot structure and prolong fibrinolysis, thereby enhancing the prothrombotic milieu (19, 37). TF, the key initiator of the coagulation cascade, is produced by various cell types with monocytes being a major source. TF expression is very low under basal conditions (38) but increases upon stimulation with cytokines (i.e., TNF-α and IL-1β) resulting in activation of the coagulation system (39–41).

Complement interacts with the fluid phase of coagulation at different levels. Mannose-associated serine protease 1 (MASP-1) exhibits a thrombin-like profile by activating fibrinogen and FXIII. It thereby leads to the formation of a crosslinked fibrin clot although the catalytic efficiency compared with thrombin is greatly reduced (42). Furthermore, MASP-1 is able to induce prothrombin and thrombin activatable fibrinolysis inhibitor (TAFI), altogether leading to the formation of thinner fibrin fibers and longer lysis time (43). Similarly, MASP-2 cleaves prothrombin to thrombin (42, 44). The C1 inhibitor not only inhibits C1r/s, MASP-1, and MASP-2 but also inhibits FXIIa and FXIa (45). Shats-Tseytlina demonstrated already in 1994 that the activation of the complement cascade leads to the formation of a prothrombotic clot with thinner fibers. Using proteomics analysis, complements C1, C3, C4, C5a, and factor B were demonstrated in plasma clots (46). Of these, C3 binds with high affinity to fibrinogen leading to a prothrombotic clot structure and prolongation of clot lysis *in vitro* (47, 48). In addition to complement C3, C5a is known to induce TF and PAI-1 expressions (49, 50). Activation of the complement system and generation of the terminal C5b–9 complex further induce endothelial cells to produce TF and vWF thereby generating a prothrombotic phenotype (51).

### Microparticles

Microparticles (MPs) are cell membrane-derived particles that can promote both coagulation and inflammation (Figure 1). Following activation or apoptosis, they are released from various cell types, including platelets, endothelial cells, red blood cells, and leukocytes. Depending on their origin they vary in size (0.2–1 μm) and their membrane composition of phospholipids and proteins. MPs can be detected in the circulation of healthy control individuals and are elevated in inflammatory situations, such as sepsis (52) and diabetes mellitus (53). MPs are directly able to modulate nitric oxide production from endothelial cells, induce cytokine release and prostacycline production as well as adherence of monocytes to the endothelium (54). The two major procoagulants found on the surface of MPs are phosphatidylserine and TF (55) thereby contributing to a prothrombotic state in various diseases. Furthermore, MPs can harbor and transport microRNA thereby impacting protein expression of target cells (56, 57).

### Neutrophil Extracellular Traps

Activation of neutrophils causes the release of web-like structures of DNA, so-called neutrophil extracellular traps (NETs). Studies demonstrated NETs to display procoagulatory properties, including the induction of platelet adhesion, aggregation, and fibrin deposition on their surface. Histones, cationic proteins that are associated with DNA, can be actively secreted by activated inflammatory cells, such as neutrophils, contributing to the formation of NETs. In this context, they have been shown to promote platelet aggregation and thrombin formation through platelet-dependent mechanisms, including platelet toll-like receptor (TLR) 2 and TLR4 (58). Bosmann and colleagues demonstrated complement C5a to be able to trigger the appearance of histones by binding to its receptors C5aR and C5L2 (59), thereby contributing to an increased inflammatory and prothrombotic milieu. A more recent study in acute STEMI demonstrated that the interaction of thrombin-activated platelets with polymorphonuclear neutrophils at the site of plaque rupture results in local NET formation and delivery of active TF (60).

## Atherosclerosis in Patients with Chronic Inflammatory Diseases

**Figure 2:**
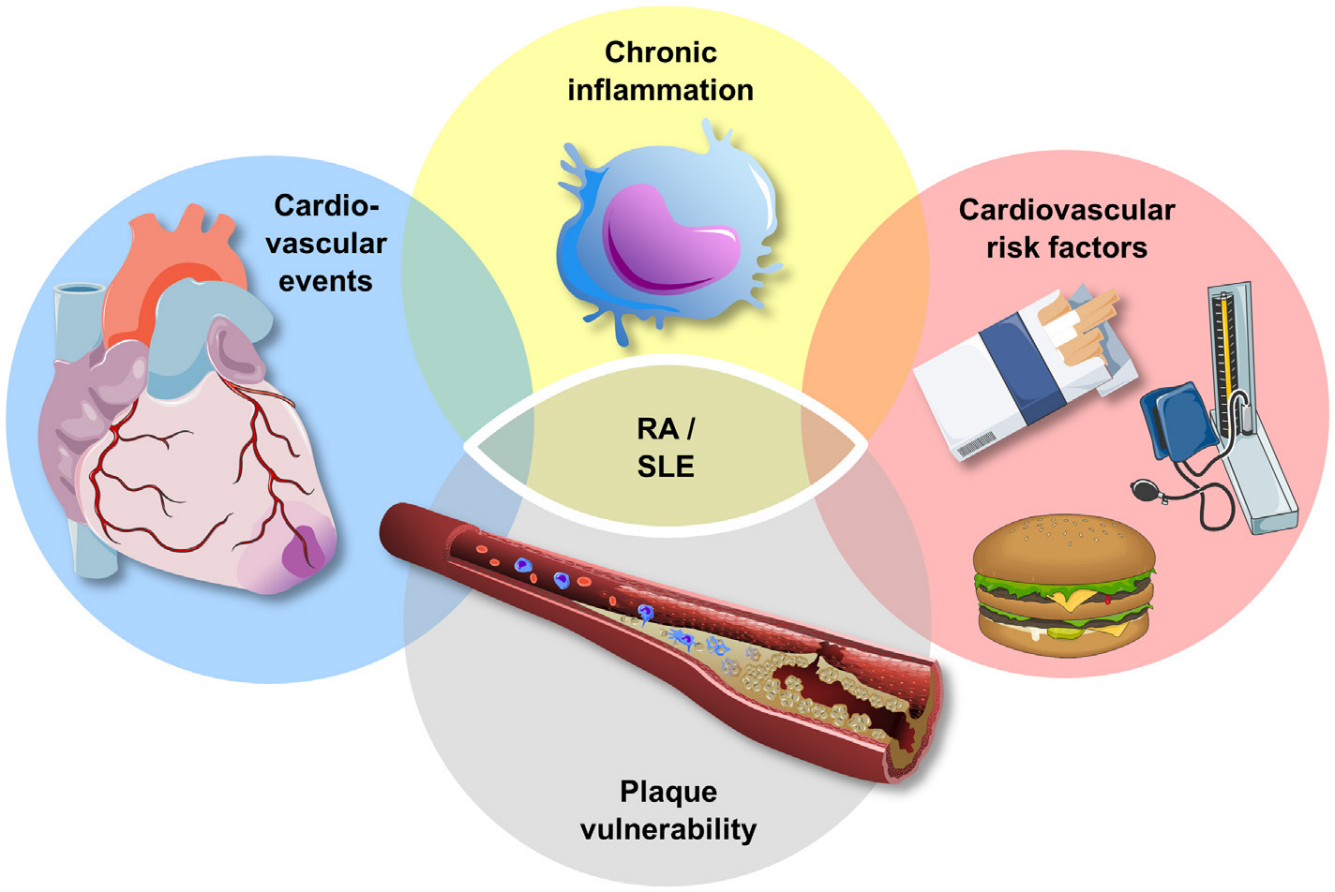
**Atherosclerosis and chronic inflammatory disease**. Patients with chronic inflammatory disease, such as rheumatoid arthritis (RA) and systemic lupus erythematosus (SLE), exhibit an increased risk for traditional cardiovascular risk factors, plaque vulnerability, and cardiovascular events. The increase in cardiovascular events in this high-risk group cannot be explained entirely by the increase in cardiovascular risk factors.

As highlighted above, atherosclerosis is characterized by an inflammatory process in the vessel wall (61). Thus, inflammatory biomarkers and cytokines, such as hsCRP, have been related with progressive cardiovascular disease as well as an increased risk for cardiovascular events (14, 62–65). On the other hand, patients with a chronic high state of inflammation, such as autoimmune diseases, including rheumatoid arthritis (RA) and systemic lupus erythematosus (SLE), are at a high risk for cardiovascular morbidity and mortality, and the increased cardiovascular risk of these patients is related to the extent of inflammation (66) (Figure 2). However, the underlying pattern, pathophysiology, and phenotype of this increased cardiovascular risk in patients with chronic inflammation vary between diseases and are discussed below for RA and SLE.

### Rheumatoid Arthritis

Patients with RA exhibit an increased cardiovascular risk with higher rates of sudden cardiac death and unrecognized MI compared with age- and sex-matched patients without RA (67). Furthermore, atherosclerosis starts early and is more diffuse over multiple vascular beds in this high-risk group (68). It could be demonstrated that patients with RA and diabetes share similar frequency and severity regarding preclinical atherosclerosis (69). An autopsy study of 41 patients with RA and 82 age- and sex-matched controls revealed a higher incidence of vulnerable plaques in the left anterior descending coronary artery and more plaque inflammation in patients with RA (70). Recently, a study by Karpouzas et al. analyzed plaque morphology using CT angiography and found a higher atherosclerotic burden in patients with RA. Interestingly, disease activity was associated with the presence of non-calcified and mixed, but not calcified plaques in this study, suggesting that disease activity is associated with plaque vulnerability (71). In light of these data, it is tempting to speculate that a more vulnerable plaque phenotype is responsible for the increased cardiovascular risk in patients with RA. Furthermore, these findings are in line with an increased prevalence of traditional cardiovascular risk factors, such as diabetes mellitus, hypertension, smoking, and obesity, in patients with RA [for review, see Ref. (72)]. However, several studies demonstrated that the cardiovascular risk in patients with RA following adjustment for classical cardiovascular risk factors remained high, suggesting that the increased risk in these patients cannot be fully explained by classical cardiovascular risk factors (67, 73). Vice versa, traditional cardiovascular risk assessment models are of limited value in patients with RA and should thus be used with caution in this high-risk group (74). Taken together, these data emphasize the link between systemic inflammation on the one hand and atherothrombosis on the other hand. However, patients with RA are not solely characterized by increased atherothrombosis but also by high rates of valvular heart disease, myocarditis, and non-ischemic cardiac failure, which all contribute to the increased cardiovascular risk in this high-risk group of patients (75).

The recognition of progressive vascular disease by the use of circulating biomarkers is a challenge in patients with a chronic high state of inflammation as conventional, inflammatory biomarkers may lack specificity in this high-risk group. Recently, we have been able to associate C-peptide, which is mainly regulated by insulin secretion and kidney function (76), to the presence of coronary artery calcification in patients with RA (77). Interestingly, the inflammatory biomarker YKL-40, which is thought to play a role in both atherogenesis and RA (62, 78–80), was a predictor for the presence of coronary artery calcification in patients with RA in this study, supporting the link between inflammation and atherosclerosis in patients with RA (Figure 2).

In addition, others found striking similarities between the pathogenesis of both RA and atherosclerosis, including endothelial activation, inflammatory cell infiltration and activation, neovascularization, and collagen degradation via matrix metalloproteinases [for review, see Ref. (72)].

### Systemic Lupus Erythematosus

Patients with SLE exhibit an increased risk for mortality (81, 82), which is in part attributed to disease activity and in part to increased cardiovascular deaths. This used to result in a bimodal mortality pattern and was described almost four decades ago (83). Presently, the mortality associated with disease activity has declined with advances in disease treatment, which results in increased life expectancy and a constant mortality rate over time (84). With these improvements in treating patients with SLE, cardiovascular and infectious complications are now the main direct cause of death among both early and late fatalities (84). Recently, a large retrospective cohort study in Taiwan compared the incidence of acute MI between patients with SLE compared to age- and sex-matched controls. The investigators found an adjusted hazard ratio of 5.11 for acute MI associated with SLE, which was even larger in the female population (hazard ratio 6.28) (85). Interestingly, patients with SLE were not only associated with a higher risk of acute MI but also with an increased mortality post-MI (85).

Similar to what is described above for patients with RA, patients with SLE exhibit an increased prevalence of traditional cardiovascular risk factors, including smoking, dyslipidemia, hypertension, and diabetes (86). However, adjustment for Framingham risk factors could not fully account for the increased cardiovascular risk in patients with SLE (87). These findings are in line with a recent meta-analysis including 17,187 patients, which found that both traditional and disease specific risk factors contribute to the risk in patients with SLE. Disease-specific risk factors included the presence of autoantibodies, neurological disorders, and to a lesser extent organ damage and SLE activity (88). Furthermore, antiphospholipid antibodies were related to mitral valve nodules and significant mitral regurgitation (89), suggesting that certain SLE-specific risk factors may also be associated with cardiovascular disease but not related to atherothrombosis.

Taken together, there appears to be a link between patients with a chronic high state of inflammation and progressive cardiovascular disease. Whereas RA and SLE demonstrate distinct immunological profiles and unique cytokine patterns [for review, see Ref. (90, 91, 75)], both share an increased cardiovascular mortality. Further studies are needed to evaluate the molecular mechanisms involved in the increased cardiovascular risk in patients with chronic inflammation and the interplay between chronic inflammation, cardiovascular risk factors, and progressive cardiovascular disease.

## Inflammation in Patients with Diabetes Mellitus

The cardiovascular vulnerability of diabetic and prediabetic patients is predictable by a variety of inflammatory markers. These are indicative of an activated innate immune system featuring elevated circulating levels of IL-1, TNF-α, IL-6, and hsCRP (92). Among these, hsCRP has gained the most attention as a predictor of diabetes, cardiovascular disease, and mortality (14, 93). In addition, low-grade inflammation is linked to activation of the coagulation system featuring increased levels of fibrinogen and PAI-1 as predisposing factor for thrombus formation (94).

A variety of mechanisms have been proposed for the initiation of subclinical inflammation in metabolic disease. Among these, obesity leads to hypertrophic adipocytes, which are unable to cope with an overwhelming supply of fatty acids (95). The consequential release of free fatty acids into the circulation causes steatosis of various organs including liver and muscle (95). Limited lipid storage capacity of their organelles results in metabolic disturbance and oxidative stress, which has been termed lipid toxicity. The consequential cellular damage and apoptosis requires debris-clearing recruitment of macrophages. These eliminate cell fragments as a prerequisite for tissue regeneration. Low-grade inflammation accompanying this cellular turnover is beneficial and part of a healing process. Persistence of pathogenic stimuli with ongoing cellular damage, however, leads to chronic inflammation and disturbed tissue integrity (96). Initiation of fibrosis may lead to an irreversible scaring process as found for steatohepatitis with transition to liver cirrhosis.

Saturated fatty acids further serve as ligands for pattern recognition receptors including TLR2 and 4 (97, 98). These provide a primitive line of defense of the innate immune system able to recognize a variety of microbial fragments and cell debris. TLRs signal downstream to various inflammatory pathways, including NF-κB and MAP-kinase, with secretion of inflammatory cytokines, including TNF-α, IL-1, and IL-6. In addition, activation of NF-κB causes direct inhibition of the insulin receptor cascade as an alternative mechanism of inflammation-mediated insulin resistance (99). Genetic or pharmaceutical inhibition of NF-κB improves glucose metabolism in mice and men. Indeed treatment with salsalate – a prodrug of salicylic acid, which inhibits NF-κB at high doses – lowers fasting glucose in non-diabetic, obese individuals (99, 100). Salsalate, however, also slightly increases plasma LDL-cholesterol levels and was ineffective in improving flow-mediated dilatation in patients with diabetes, questioning its clinical utility (100). Another anti-inflammatory strategy in metabolic disease, which has advanced to the clinics, is blockade of the IL-1 receptor system. This improved glucose metabolism and insulin sensitivity in small clinical studies and was associated with a reduction of inflammatory serum parameters (101). Blockade of the IL-1 system is currently evaluated in a large cardiovascular endpoint study (CANTOS) in which the IL-1-directed antibody canakinumab is tested versus placebo in a randomized trial in 17,200 stable, post-MI patients with persistent high CRP levels (102). A dedicated sub-study further investigates the effect of IL-1 system blockade on glucose metabolism and insulin resistance with trial termination being expected in 2017. The efficacy of inflammation targeting therapies in metabolic disease is further investigated in the Cardiovascular Inflammation Reduction Trial (CIRT) using low-dose methotrexate (target dose 15–20 mg/week) treatment. This study randomizes 7000 prior MI patients with either type 2 diabetes or the metabolic syndrome with an average follow-up period of 3–5 years expecting termination in 2018 (103).

More recently, metabolic inflammation has further been attributed to a disturbed intestinal barrier function (104). Increased circulating concentrations of endotoxin are detected in patients with obesity and diabetes with direct associations to cardiovascular disease (105). A disturbed intestinal barrier enables the passive diffusion of bacterial fragments and lipopolysaccarides across the intestinal mucosa. Interestingly, this seems to be modulated by dietary factors with high nutritional fat intake promoting endotoxin absorption (106). Circulating endotoxin serves as a ligand for TLR2 and 4 with activation of the innate immune system. Chronic low-dose endotoxin application was consequently found to cause obesity and insulin resistance in rodent models while knockout of TLR4 prevented diet-induced obesity, glucose intolerance, and atherosclerosis (107–109). Furthermore, mice lacking the functional LPS receptor CD14 were resistant to diet-induced obesity and related disorders (110) while antibiotic eradication of the gut flora led to reduced metabolic inflammation, insulin resistance, and fat mass development (111). In consequence, germ-free mice were protected from high-fat diet-induced inflammation (112). Modulation of the intestinal microbiome as a source for endotoxin generation might therefore provide a new treatment strategy for diabetes and cardiovascular disease prevention.

These observations demonstrate a close interaction between the immune system and metabolism which is particularly present in patients with diabetes. Activation of the immune system does thereby not depend on an external stressor but is the consequence of overnutrition as an endogenous stressor. Concepts of anti-inflammatory therapies might prove effective in improving diabetes and cardiovascular outcome.

## Conclusion

Over the last 30 years, inflammation has increasingly been recognized as an important pathophysiological link between vascular disease, atherothrombosis, metabolic disorders, and chronic autoimmune diseases. A plethora of mediators, such as cytokines, chemokines, and adipokines, released from various cells in different organs contribute to both local and remote stimulations of inflammatory cells, thus creating a network of interactions at different levels in the organism. Future therapeutic strategies need to be developed to examine how and by which tools this interaction can be targeted to modulate cardiovascular disease in high-risk patients.

## Conflict of Interest Statement

The authors declare that the research was conducted in the absence of any commercial or financial relationships that could be construed as a potential conflict of interest.
